# Potent Broad-Spectrum Antibacterial Activity of Amphiphilic Peptides against Multidrug-Resistant Bacteria

**DOI:** 10.3390/microorganisms8091398

**Published:** 2020-09-11

**Authors:** Yuan Liu, Jingru Shi, Ziwen Tong, Yuqian Jia, Kangni Yang, Zhiqiang Wang

**Affiliations:** 1 College of Veterinary Medicine, Yangzhou University, Yangzhou 225009, China; liuyuan2018@yzu.edu.cn (Y.L.); Shijr2019@163.com (J.S.); tongzw2019@163.com (Z.T.); jiayq2019@163.com (Y.J.); yangkn2019@163.com (K.Y.); 2 Institute of Comparative Medicine, Yangzhou University, Yangzhou 225009, China; 3 Jiangsu Co-Innovation Center for Prevention and Control of Important Animal Infectious Diseases and Zoonoses, Yangzhou University, Yangzhou 225009, China; 4 Joint International Research Laboratory of Agriculture and Agri-Product Safety, The Ministry of Education of China, Yangzhou University, Yangzhou 225009, China

**Keywords:** antimicrobial peptides, antibiotic resistance, colistin, tigecycline, Gram-negative bacteria

## Abstract

The emergence and prevalence of multidrug-resistant (MDR) bacteria particularly Gram-negative bacteria presents a global crisis for human health. Colistin and tigecycline were recognized as the last resort of defenses against MDR Gram-negative pathogens. However, the emergence and prevalence of MCR or Tet(X)-mediated acquired drug resistance drastically impaired their clinical efficacy. It has been suggested that antimicrobial peptides might act a crucial role in combating antibiotic resistant bacteria owing to their multiple modes of action and characteristics that are not prone to developing drug resistance. Herein, we report a safe and stable tryptophan-rich amphiphilic peptide termed WRK-12 with broad-spectrum antibacterial activity against various MDR bacteria, including MRSA, colistin and tigecycline-resistant *Escherichia coli*. Mechanistical studies showed that WRK-12 killed resistant *E. coli* through permeabilizing the bacterial membrane, dissipating membrane potential and triggering the production of reactive oxygen species (ROS). Meanwhile, WRK-12 significantly inhibited the formation of an *E. coli* biofilm in a dose-dependent manner. These findings revealed that amphiphilic peptide WRK-12 is a promising drug candidate in the fight against MDR bacteria.

## 1. Introduction

Pathogenic bacteria particularly Gram-negative pathogens such as *Escherichia coli*, *Klebsiella pneumoniae*, *Pseudomonas aeruginosa* and *Acinetobacter baumannii* have been a major cause of systemic infections in clinics [1,2]. More alarmingly, these pathogens have developed multiple acquired resistance against current antimicrobial treatments [3,4,5]. For example, the acquisition of metallo-β-lactamases (MBLs) such as NDM-1 resulted in the emergence of carbapenem-resistant Gram-negative pathogens [6]. Mobile colistin resistance gene *mcr-1* and its variants [7,8] protect Enterobacteriaceae from colistin killing, an important cationic antibiotic that is recognized as one of last-resort options against multidrug-resistant (MDR) Gram-negative bacteria [9]. In addition, recent study revealed that *tet*(X3/X4) genes located on plasmid confer high level tigecycline resistance in *E. coli* and *A. baumannii* [10,11]. To date, these drug-resistant pathogens have been classified as a critical priority for global human health by the WHO (World Health Organization). Notably, the co-harboring of *bla*_NDM_, *mcr-1* and/or *tet*(X) genes in clinical isolates makes it more difficult to treat MDR pathogens’ associated infectious diseases. There is an urgent and unmet need to identify new antimicrobial agents to address this resistance crisis.

Antimicrobial peptides (AMPs), also named host defense peptides, are originated from various organisms and are important immune modulation molecules that protect the host from the invading pathogens [12]. Compared with conventional antimicrobial agents, AMPs possess distinct modes of action and are less likely to achieve resistance [13,14]. However, the high cost of peptides, the lability to proteases, less bioavailable and nonspecific toxicity still hinder these AMPs in clinical practice. A more rational design of AMPs contributes to improving their stability and antibacterial activity. One strategy is to introduce more aromatic amino acids such as tryptophan into peptides. The interaction of highly hydrophobic indole ring of tryptophan with the headgroup region of the membrane enable the tryptophan-rich peptides to greatly penetrate the cytoplasmic membrane [15,16]. In addition, pairwise Trp-Trp interactions lead to a distinctive cross-strand contact and stable tertiary structure [17]. A typical example is a synthetic hexapeptide MP196 (RWRWRW-NH_2_), which is effective against Gram-positive bacteria but displays relatively weak antibacterial activity against Gram-negative bacteria [18]. However, the high hemolysis to RBCs and low in vivo stability strongly limit its utilization in the clinical setting [19]. Nevertheless, MP196 provides a promising parent structure for the next structural optimization or derivatization, and has yielded several AMPs with better pharmacological properties, stability and/or improved activities [20,21].

To identify novel leads against MDR bacteria, we collected and designed a collection of tryptophan-rich amphiphilic antibacterial peptides based on the structure of MP196 and assessed their antibacterial activity against a panel of MDR bacteria. As a consequence, we found a stable and potent dodecapeptide termed WRK-12 that could effectively kill various MDR bacteria, including notorious methicillin-resistant *Staphylococcus aureus* (MRSA), vancomycin resistant Enterococcus (VRE), carbapenem-resistant Enterobacteriaceae (CRE) and *mcr-1*-positive *E. coli* (MCRPEC) and *tet*(X4)-positive bacteria. Mechanistical experiments indicated that WRK-12 permeabilizes the bacterial membrane and triggers the production of reactive oxygen species (ROS). Consistently, the addition of ROS scavenger NAC (*N*-acetylcysteine) abolishes its antibacterial activity. Moreover, WRK-12 dramatically prevented the formation of biofilm, thus providing a lead compound in the battle against MDR bacteria.

## 2. Materials and Methods

### 2.1. Peptide Synthesis and Validation

All peptides used in this study were synthesized by GL Biochem (Shanghai, China) using a solid phase peptide synthesis (SPPS) method [22], and their accurate molecular weights were determined by matrix-assisted laser desorption/ionization time-of-flight mass spectrometry (MALDI-TOF MS). The peptide purity (>95%) and retention time were determined by reversed-phase high-performance liquid chromatography (HPLC).

The charge and hydrophobic moment of all peptides were calculated using the HeliQuest analysis website (http://heliquest.ipmc.cnrs.fr/cgi-bin/ComputParamsV2.py). The three-dimensional structure projection of three active peptides was predicted by I-TASSER (http://zhanglab.ccmb.med.umich.edu/I-TASSER/).

### 2.2. Bacteria Strains and Reagents

All bacteria strains including reference strains and clinical isolates used in this study have been listed in Table S1. All antibiotics were obtained from the China Institute of Veterinary Drug Control. Other chemical compounds were purchased from TCI (Shanghai, China)

### 2.3. Antibacterial Activity Tests

Minimum inhibitory concentrations (MICs) of peptides were determined by broth micro-dilution according to the CLSI2018 guideline [23]. Briefly, overnight strains were diluted 1:100 into Mueller–Hinton broth (MHB) and incubated at 37 °C with sharking at 200 rpm for 4 h. Subsequently, varying concentrations of drugs were mixed with an equal volume of bacterial suspensions in MHB containing approximately 1.5 × 10^6^ colony-forming units per mL in a sterilized 96-well microtitre plate (Corning). After 18 h of incubation at 37 °C, MIC values were determined as the lowest concentrations of drugs with no visible growth of bacteria. For the minimum bactericidal concentration (MBC) assay, 50 μL of mixture was taken out from clear well in MIC assays, resuspended in fresh media and plated onto Mueller–Hinton agar (MHA) overnight at 37 °C. The MBC is defined as the lowest concentration of drugs that killed 99.9% of the bacterial cells. Experiments were performed with two biological replicates.

Salts and serum stability. For assessing the effect of salts and serum on the activity of WRK-12, three salts (including Na^+^, K^+^, Ca^2+^, final concentration 10 mM), 10% fetal bovine serum (FBS) and Dulbecco’s Modified Eagle Medium (DMEM) were added into MHB for a following MIC assay.

Thermal, pH and proteolytic stability. WRK-12 was preincubated at different temperatures (from 25 to 121 °C), pH (from 2 to 12) or proteases (pepsin, trypsin and papain, final concentration 10 mg/mL) for 1 h. Samples after pH treatment were readjusted to pH = 7.2 to determine the residual antibacterial activity by MIC tests. Samples after proteases treatment were heated at 80 °C for 30 min, and centrifuged at 13,000× *g* for 30 min to precipitate proteases, and the residual antibacterial activity of supernatants was tested by MIC analysis.

Lipopolysaccharides (LPS), lipids or NAC inhibition assay. The MICs of WRK-12 in the presence of increasing concentrations of lipopolysaccharide (LPS, 0 to 128 μg/mL) from *E. coli* O111:B4 (Sigma) or various lipids (including phosphatidylcholine (PC), phosphatidylethanolamine (PE), phosphatidylglycerol (PG) and cardiolipin (CL) (0 to 16 μg/mL, sigma) or NAC (*N*-acetylcysteine, 0 to 2.5 mM) against *E. coli* 1F28 were determined as described above. Experiments were performed with biological replicates.

### 2.4. Hemolysis Analysis

Hemolytic activity of all peptides was evaluated based on previous studies [24,25]. Sheep red blood cells (RBCs) were washed with 0.9% saline two times, re-suspended to obtain 8% red blood cell suspension and then mixed with increasing concentrations of AMPs at 37 °C for 1 h. Sterilized PBS and double-distilled water (ddH_2_O) were used as blank and positive control, respectively. The absorption of released hemoglobin was measured at 576 nm by an Infinite M200 Microplate reader (Tecan, Männedorf, Switzerland). Hemolysis rate was calculated by comparing the absorbance of the sample and the positive control after subtracting the blank control.

### 2.5. CD Measurements

Circular dichroism (CD) spectra of the peptides were recorded with a J-810 spectropolarimeter (Jasco, Tokyo, Japan) at 25 °C. The spectra were measured in 0.01 M PBS, 50 μM LPS or 50 mM SDS (final concentration, 100 μg/mL). The CD spectra were recorded at a wavelength of 190 to 260 nm, and the data are expressed as mean residue ellipticity

### 2.6. Outer Membrane Permeabilization

*E. coli* 1F28 were grown overnight at 37 °C with shaking at 200 rpm. Bacterial cells were washed and resuspended with 5 mM HEPES to an OD_600_ of 0.5 and incubated with fluorescent probe 1-*N*-phenylnaphthylamine (NPN, 0.1 μM) at 37 °C in a humidified atmosphere for 30 min [26]. Subsequently, 190 μL of probe-labelled cells were mixed with 10 μL WRK-12 (0 to 128 μg/mL) or colistin as a positive control (128 μg/mL) in a sterile 96-well black plate. After incubation at 37 °C in a humidified atmosphere for 60 min, fluorescence intensity was measured on an Infinite M200 Microplate reader (Tecan, Männedorf, Switzerland) (λexcitation = 350 nm, λemission = 420 nm).

### 2.7. Membrane Permeability Assay

*E. coli* 1F28 cells at exponential growth phase (OD_600_ = 0.5) were incubated with propidium iodide (PI, 0.5 μM, Beyotime), followed by the addition of WRK-12 (0 to 128 μg/mL) or colistin (128 μg/mL). After incubation for 60 min as described above, fluorescence intensity (λexcitation = 535 nm, λemission = 615 nm) was measured using a Microplate reader (Tecan, Männedorf, Switzerland).

### 2.8. Cytoplasmic Membrane Potential

A fluorescent probe DiSC_3_(5) (Aladdin, Shanghai, China) was utilized to evaluate the effect of WRK-12 on bacterial membrane potential [27]. *E. coli* 1F28 cells were probed with 3,3′-dipropylthiadicarbocyanine iodide (DiSC_3_(5), 0.5 μM) for 30 min, and then treated with WRK-12 (0 to 128 μg/mL) or colistin (128 μg/mL) for 60 min. Subsequently, the dissipated membrane potential was determined by monitoring the fluorescence intensity (λexcitation = 622 nm, λemission = 670 nm) using a Microplate reader (Tecan, Männedorf, Switzerland).

### 2.9. ROS Measurements

2′,7′-Dichlorodihydrofluorescein diacetate (DCFH-DA, 10 μM) was incubated with *E. coli* 1F28 cells for 30 min. After washing with 0.01 M PBS, the probed cells were mixed with WRK-12 (0 to 128 μg/mL) or colistin (128 μg/mL) for 60 min. The ROS levels were assessed by monitoring the fluorescence intensity (λexcitation = 488 nm, λemission = 525 nm) using a Microplate reader (Tecan, Männedorf, Switzerland).

### 2.10. Prevention of Biofilm Formation

The prevention of biofilm formation was assessed as described previously [28]. Briefly, bacteria (1 × 10^5^ CFUs per mL) were exposed to WRK-12 solutions (with final concentrations ranging from 0.25 to 32 μg/mL). As an untreated control, bacteria were exposed to MHB without drugs. After 24 h incubation at 37 °C in a humidified atmosphere, planktonic bacteria were removed by phosphate buffer solution (PBS). Biofilms were fixed with methanol for 15 min, then sucked out the fixative and air dry naturally. Then, biofilms were stained with 0.1% crystal violet for 15 min, washed and dried naturally. Finally, 33% acetic acid was used to dissolve crystal violet. The optical density at 570 nm was determined as a measure of biofilm mass.

### 2.11. Statistical Analysis

All data were shown as mean ± SD from at least three triplicates. Statistical analysis was performed using GraphPad Prism 8 (* *p* < 0.05, ** *p* < 0.01, *** *p* < 0.001).

## 3. Results and Discussion

### 3.1. Characterizations of Engineered Peptides

A collection of tryptophan-rich linear peptides on the basis of MP196 (RWRWRW-NH_2_) was designed by increasing peptide length or positive charge or hydrophobicity, replacing amino acids and/or *N*-terminal acetylation (Table 1). These peptides were synthesized via solid-phase peptide synthesis (SPPS), purified by reverse-phase high-performance liquid chromatography (RP-HPLC) (Figure S1) and validated by matrix-assisted laser desorption/ionization time-of-flight mass spectrometry (MALDI-TOF MS) (Figure S2). Chemical information and characterization of these engineered peptides are shown in Table 1. All peptides were accurately obtained with the purifies higher than 95%, and the molecular weights calculated by mass spectrometry are consistent with their theoretical molecular mass value, suggesting that all the engineered peptides were successfully synthesized. All engineered peptides belong to cationic AMPs, with 3 or 6 net charges. The percentage of acetonitrile at RP-HPLC elution was regarded as a relative measure of peptides’ hydrophobicity. Accordingly, the majority of engineered peptides, except WK-6, exhibited higher hydrophobicity than MP196. The hydrophobicity order was listed as follows: WRK-12 > WL-9 > WR-6 > WV-9 > KW-6 > WKK-12 > WR-12 > MP196 > WK-6.

### 3.2. Broad-Spectrum Antibacterial Activity of Engineered Peptides In Vitro

Subsequently, we assessed the antibacterial activity of these peptides against a multidrug-resistant (MDR) strain *E. coli* B2, which is almost resistant to all clinically used antibiotics. MIC results (Table 1) revealed that three repeats of dipeptide such as MP196, WR-6 (WRWRWR-NH_2_), KW-6 (KWKWKW-NH_2_) and WK-6 (WKWKWK-NH_2_) have no inhibitory effect on *E. coli* B2 (MIC > 64 μg/mL). The introduction of hydrophobic Leucine (Leu, L) or Valine (Val, V) leads to two new nonapeptides WL-9 (WRLWRLWRL-NH_2_) and WV-9 (WKVWKVWKV-NH_2_). Interestingly, WL-9 exerted modest antibacterial activity (MIC, 8 μg/mL), whereas WV-9 is inactive (MIC > 64 μg/mL).

It has been suggested that cationicity and acetylation at *N*-terminal contribute to improve the activity of AMPs [29]; Therefore, three positive amino acids and/or acetylation at *N*-terminal were further introduced on the basis of two nonapeptides. As a consequence, we constructed three novel dodecapeptides including WR-12, WRK-12 and WKK-12 with acetylation at the *N*-terminus and amidation at the *C*-terminus. As expected, insertion of three positive amino acids in WL-9 (produce WR-12 or WRK-12) significantly increased its antibacterial activity with MICs decreasing by 4-fold change.

**Figure 1:**
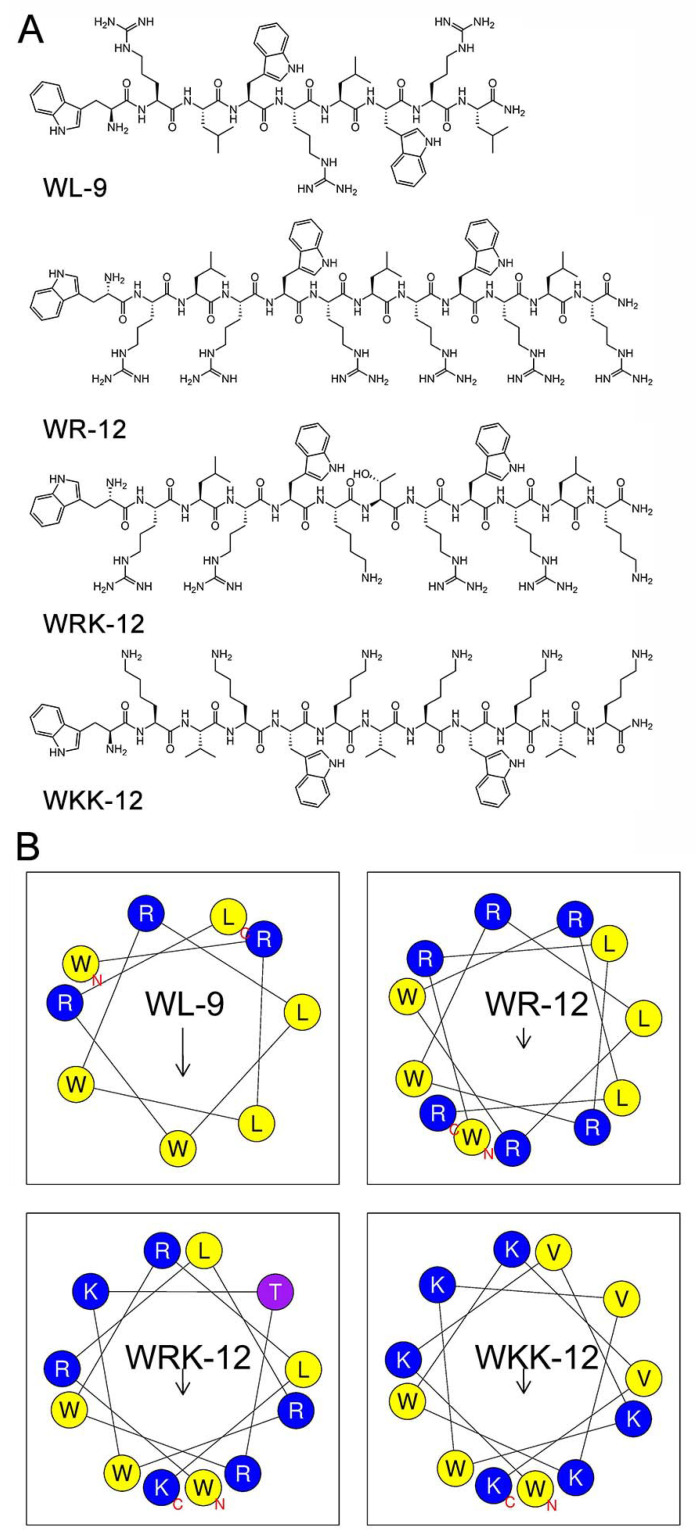
Design of tryptophan-rich amphiphilic antibacterial peptides. (**A**) Chemical structures of four active AMPs (WL-9, WR-12, WRK-12 and WKK-12). (**B**) Helical wheel projections of four AMPs. The potentially charged residues, hydrophobicity residues and uncharged residues were marked as blue, yellow and pink, respectively. The longer the arrow length, the greater the relative hydrophobic moments in the figure.

The wheel diagram showed these four active AMPs including WL-9, WR-12, WRK-12 and WKK-12 (Figure 1A), which exhibited imperfect amphiphilic structures that possessed interrupted hydrophobic and cationic faces (Figure 1B). Interestingly, although WRK-12 displayed high hydrophobicity, the solubility test showed that only WRK-12 has the highest solubility in 0.01 M PBS or water, whereas the other three peptides are slightly soluble in water. The introduction of Threonine (Thr, T) in WRK-12, an uncharged polar amino acid, may account for this paradox. Prior studies have illustrated that *N*-terminus acetylation has a critical effect on both peptide secondary structure and penetration ability on the bacterial membrane, as well as its in vivo efficacy [30,31]. Considering these points, WRK-12 and WKK-12 were chosen as potential candidates for our next study.

MIC tests showed that WRK-12 and WKK-12 displayed antibacterial activity against MDR *E. coli* B2 with MIC values of 2 and 64 μg/mL, respectively. Subsequently, we investigated the antibacterial spectrum of WRK-12 and WKK-12 in a panel of MDR bacterial isolates. As shown in Table 2, we found that WRK-12 showed the broad-spectrum antibacterial activity against all test strains with MIC values from 2 to 4 μg/mL and MBC values from 2 to 8 μg/mL. These strains include hard-to-treat Gram-positive bacteria such as MRSA and VRE, MDR Gram-negative bacteria that are resistant to the last resort of clinically available antibiotics such as carbapenems, colistin and tigecycline. Specifically, for both *mcr-1* and *bla*_NDM-5_ carrying colistin and carbapenems-resistant *E. coli* B2 (MIC of colistin, 8 μg/mL) and *tet*(X4)-positive tigecycline *E. coli* B3-1 (MIC of tigecycline, 32 μg/mL), WRK-12 also displayed potent activity, suggesting that the activity of WRK-12 is independent of these two resistance mechanisms. Considering the urgent need of novel antimicrobial agents for recent reported tigecycline-resistant Gram-negative bacteria in clinic, we next focused our insight on these strains. We investigated the activity of WRK-12 and WKK-12 against 11 tigecycline-resistant clinical isolates from a swine farm in 2019 (Table 3). Excitingly, WRK-12 displayed great antibacterial activity for all test strains, including important foodborne pathogen *Shigella*.

**Figure 2:**
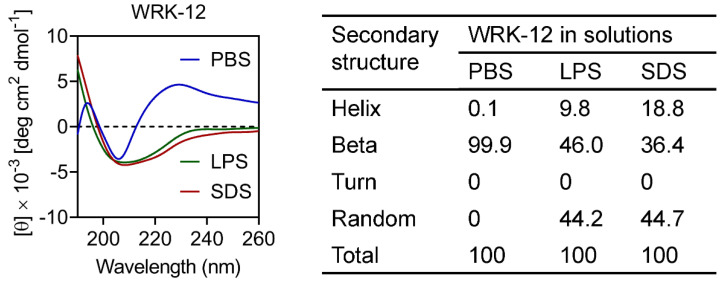
Circular dichroism (CD) spectra of WRK-12 in PBS (10 mM, pH 7.4), Lipopolysaccharide (LPS) (50 μM) and SDS (50 mM). The values from three scans were averaged per sample, and the peptide concentrations were fixed at 100 μg/mL.

The secondary structure of the engineered peptides was tested by CD spectroscopy. As shown in Figure 2, WRK-12 exhibited a complete beta sheet conformation in phosphate buffer solution (PBS). LPS is one of important components in the Gram-negative bacterial outer membrane [32], and sodium dodecyl sulfate (SDS) micelle solution was used that mimicked negatively charged prokaryotic membrane-comparable environments. In the presence of 50 μM LPS and 50 mM SDS, the proportion of random and α-helical WRK-12 increased. This result indicated that the secondary structure of WRK-12 would present in hybrid form as it interacts with the bacterial membrane.

### 3.3. A Desirable Safety and Stability of WRK-12 against Bacteria

**Figure 3:**
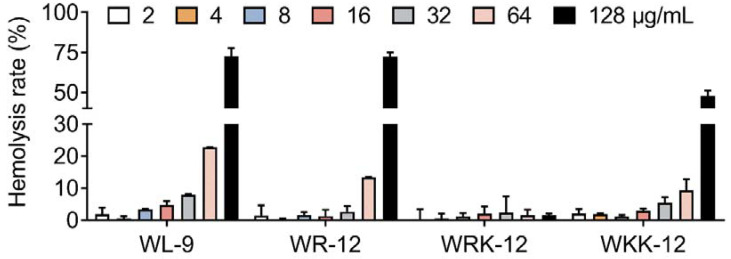
Hemolytic activity of four active peptides against mammalian red blood cells (RBCs). Sterilized PBS (10 mM) and ddH_2_O were used as a negative control and positive control, respectively.

The hemolytic activity and instability of peptides are important challenges that hinder the clinical application of drugs [33]. In our study, hemolytic analysis showed that WRK-12 had the dispensable and lowest hemolytic activity (<5% at 128 μg/mL) on mammalian RBCs compared with the other three active AMPs (Figure 3), indicating higher selectivity of WRK-12 for bacteria other than mammalian cells. Although WR-12 showed comparable antibacterial activity with WRK-12, it displayed more than 50% of the hemolytic rate at 128 μg/mL.

Great stability of antimicrobial peptides is a critical prerequisite for its in vivo efficacy. Thus, we evaluated the antibacterial activity of WRK-12 and WKK-12 against tigecycline resistant *E. coli* 1F28 and 1A34 in the presence of salt ions, serum and Dulbecco’s Modified Eagle’s Medium (DMEM) (Table 4). There was not any loss of activity in the presence of monovalent cations (Na^+^ and K^+^), whereas divalent cation Ca^2+^, 10% serum or DMEM mildly reduced the activity of WRK-12 with the MICs increasing by 2 to 4-fold. The weakened activity by the divalent cation indicated that the action of WRK-12 may be correlated with membrane damage, because the outer membrane of Gram-negative bacteria could be stabilized with divalent cations particularly Ca^2+^ and Mg^2+^ [34]. By contrast, Na^+^, Ca^2+^, 10% serum or DMEM sharply impaired the weak antibacterial effect of WKK-12 against two tested isolates.

**Figure 4:**
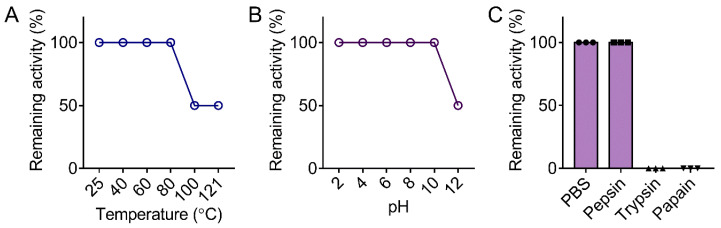
Thermal, pH and proteolytic stability of WRK-12 against E. coli 1F28. Remaining activity (%) of WRK-12 against E. coli 1F28 after treatment with varying temperature (**A**, from 25 to 121 °C), pH (**B**, from 2 to 12) and three proteases (**C**).

In addition, medium containing 10% serum and DMEM was used to simulate an in vivo matrix environment. Only 2 to 4-fold increase of MIC values were found in these conditions. Next, we also assessed the thermal, pH and proteolytic stability of WRK-12. Surprisingly, WRK-12 completely retained its activity after treatment under 100 °C or pH (2 to 10) for 1 h (Figure 4A,B), indicating that WRK-12 possesses great thermal and pH resistance. In contrast, a 50% activity reduction after exposure to 121 °C or alkaline environment (pH = 12) was observed. In the proteolytic stability study, we found that WRK-12 was resistant to pepsin treatment, but sensitive to trypsin and papain (Figure 4C). High percentage of cationic amino acids in WRK-12 may account for this result. Together, these data suggested the great salt ions, serum, thermal and pH stability of WRK-12.

### 3.4. WRK-12 Targets LPS and Bacteria-Specific Phospholipids

**Figure 5:**
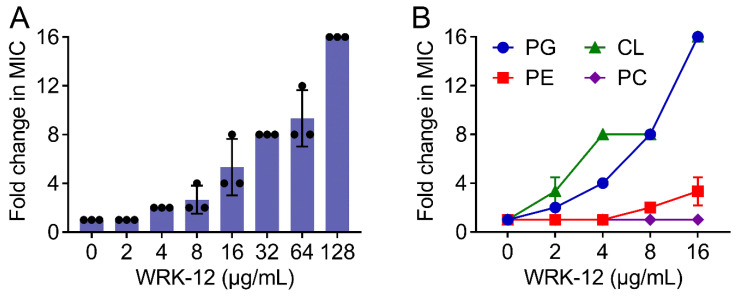
WRK-12 targets LPS and various phospholipids of the bacterial membrane. (**A**) Exogenous LPS from *E. coli* O111:B4 dose-dependent decreases the antibacterial activity of WRK-12 against *E. coli* 1F28, determined by chequerboard broth microdilution tests. (**B**) Exogenous lipids including PG, CL and PE except PC increase the MIC values of WRK-12 against *E. coli* 1F28, determined by chequerboard broth microdilution tests.

Having shown the activity and stability of WRK-12, we set out to elucidate its bactericidal targets. Previous studies have demonstrated that cationic AMPs such as MSI-78 and LL-37 can disrupt the bacterial lipid bilayer structure through their electrostatic interactions with the polar headgroups [35,36]. Considering that WRK-12 is a cationic antibacterial peptide, we hypothesize that WRK-12 may damage the bacterial membrane through targeting the specific components of the bacterial membrane. To test this, we determined the effect of exogenous LPS or phospholipid supplements on the antibacterial activity of WRK-12 against *E. coli* 1F28. Consequently, we found that addition of LPS weakened WRK-12 activity in a dose-dependent manner (Figure 5A), suggesting that LPS is a potential target of WRK-12. In addition to LPS, phospholipids including PE, phosphatidylglycerol (PG) and cardiolipin (CL) are important components of the bacterial plasma membrane, whereas phosphatidylcholine (PC) only presents in mammalian cell membranes [37]. Thus, we next performed a phospholipid competitive inhibition assay to assess the effect of exogenous lipids on WRK-12 activity. As a result, PG and CL drastically increased the MIC values of WRK-12 (16-fold at 16 μg/mL PG or CL), PE mildly impaired WRK-12 activity and no MIC changes were observed for CL (Figure 5B). These results implied that WRK-12 exerted the activity through binding to LPS in the outer membrane, as well as PG and CL that were located in the bacterial cytoplasmic membrane. Meanwhile, the notion that PG and CL have a lower proportion in mammalian cell membranes give an explanation on its high selectivity on bacteria. Nevertheless, direct binding affinity assays between WRK-12 and these potential targets are still required to further strengthen these findings.

### 3.5. WRK-12 Increases Membrane Permeability, Dissipates Membrane Potential and Induces ROS Production

**Figure 6:**
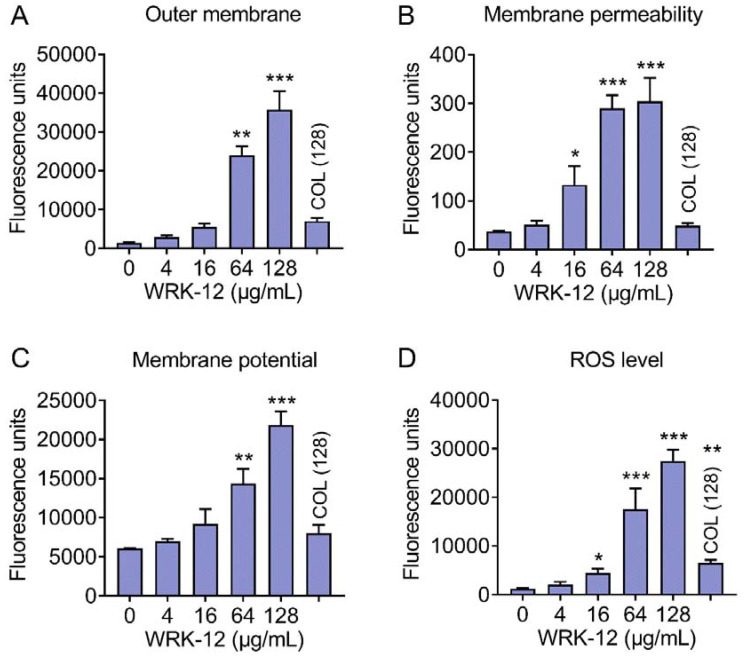
Bactericidal mechanisms of WRK-12 against multidrug resistant *E. coli.* (A and B) WRK-12 increases outer membrane and cytoplasmic membrane permeability in a dose-dependent manner. Permeability of outer membrane (**A**) and the whole membrane permeability (**B**) were assessed with fluorescence probes 1-*N*-phenylnaphthylamine (NPN, excitation 350 nm and emission 420 nm) and propidium iodide (PI, excitation 535 nm and emission 615 nm), respectively, after exposure to WRK-12 or colistin (128 μg/mL) for 1 h. (**C**) WRK-12 dissipates membrane potential in *E. coli*, probed by monitoring fluorescence intensity of 3,3′-dipropylthiadicarbocyanine iodide (DiSC3(5), excitation 622 nm and emission 670 nm). (**D**) WRK-12 triggers the production of ROS, determined by 2′,7′-dichlorodihydrofluorescein diacetate (DCFH-DA, excitation 488 nm and emission 525 nm). All data were presented as mean ± SD, and significance was determined by non-parametric one-way ANOVA. * *p* < 0.05, ** *p* < 0.01, *** *p* < 0.001. Colistin (128 μg/mL) was used as a control.

**Figure 7:**
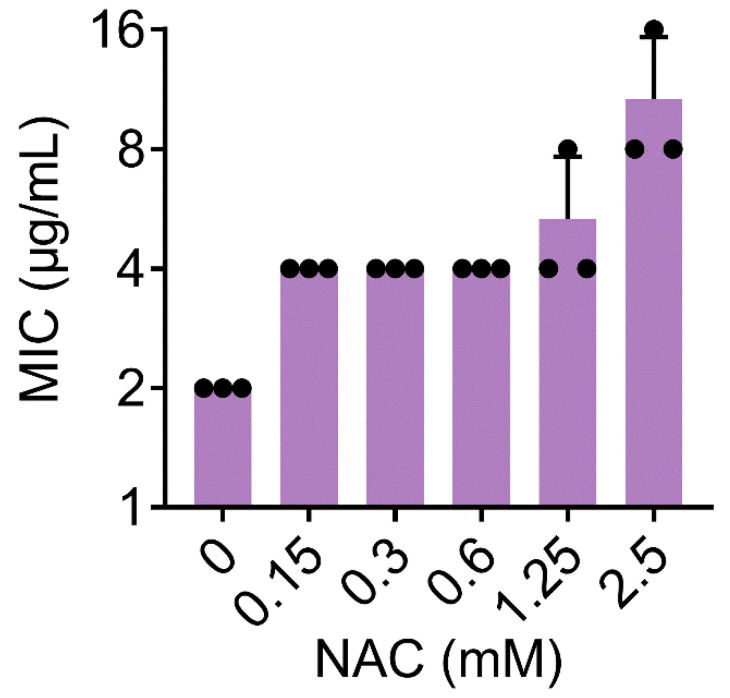
ROS scavenger NAC abolishes the antibacterial activity of WRK-12. MIC values of WRK-12 against *E. coli* 1F28 in the presence of increasing concentrations of NAC (*N*-acetylcysteine) were determined. Data were presented as mean ± SD from three independent experiments.

To further elucidate membrane damage caused by WRK-12, we used a fluorescence probe 1-*N*-phenylnaphthylamine (NPN) to assess the effect of WRK-12 on the outer membrane permeability of *E. coli*. As shown in Figure 6A, WRK-12 at 128 μg/mL caused a significant fluorescence release, which was five-fold higher than colistin (128 μg/mL), indicating that WRK-12 strongly disrupted outer membrane permeability than colistin. Then, nucleic acid fluorescent dye propidium iodide (PI) was employed to evaluate the whole membrane permeability. As a result, WRK-12 led to a dose-dependent increase of PI fluorescence, implying a remarkable damage to the bacterial membrane (Figure 6B). By contrast, colistin (128 μg/mL) showed a week fluorescence increase, similar to 4 μg/mL WRK-12. Consistently, membrane disruption has been suggested as one of crucial mechanisms of action for AMPs killing [38]. Besides, WRK-12 significantly dissipated membrane potential (ΔΨ) (Figure 6C), which was critical component of bacterial proton motive force [39]. ROS mediated killing has been evidenced to be important for bactericidal antibiotics [40]. Consistently, WRK-12 triggered the production of ROS in a concentration-dependent manner (Figure 6D). In agreement with this observation, ROS scavenger NAC abolished the antibacterial activity of WRK-12 with MICs increased by 6-fold at 2.5 mM (Figure 7), suggesting that production of ROS is crucial for WRK-12 activity against MDR pathogens.

### 3.6. WRK-12 Inhibits Biofilm Formation

**Figure 8:**
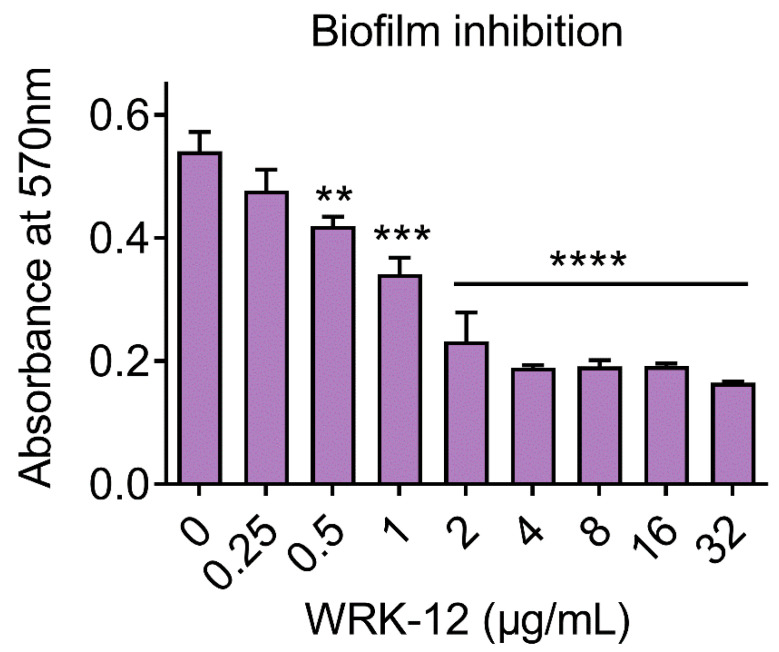
WRK-12 inhibits the formation of E. coli 1F28 biofilm in a concentration-dependent manner. Data were shown as mean ± SD from three independent experiments. ** *p* < 0.01, *** *p* < 0.001, **** *p* < 0.0001, determined by non-parametric one-way ANOVA.

Biofilms produced by bacteria play a critical role in its pathogenicity and the development of drug resistance and have been implicated in chronic infections. Recent study has highlighted the possible use of AMPs to prevent biofilm formation or to treat established biofilms [28]. Intriguingly, WRK-12 dose-dependently inhibited the formation of biofilms by tigecycline-resistant *E. coli* 1F28 (Figure 8). Notably, a significant inhibition effect of WRK-12 on biofilm formation could be observed even though at a low concentration of drug (0.5 μg/mL). The biofilm mass, as measured using crystal violet staining after 24 h, drastically reduced after exposure to 2 μg/mL or higher concentrations of WRK-12. These results indicated that WRK-12 has a beneficial effect on the inhibition of biofilm formation.

## 4. Conclusions

The emergence and prevalence of MDR pathogens call for novel and effective antimicrobial agents. In this study, we designed a series of tryptophan-rich amphiphilic peptides and investigated their antibacterial effect on MDR bacteria. A stable and potent dodecapeptide termed WRK-12 was identified, which exerts excellent activity against various pathogenic bacteria including carbapenems, colistin and tigecycline-resistant Gram-negative bacteria. Furthermore, WRK-12 displays low hemolytic activity and high salt or serum stability. Mechanical studies revealed that WRK-12 causes membrane damage and over-production of ROS through targeting LPS and bacterial-specific lipids. Collectively, the discovery of potent antimicrobial leads offers a novel therapeutic strategy to combat the increasing MDR bacteria.

## Figures and Tables

**Table 1:** Design of tryptophan-rich amphiphilic peptides and their key physicochemical parameters.

Name	Sequence (*N* → *C*)	Formula	MW	Net Charge	pI ^*a*^	H ^*b*^	Purity (%)	MIC ^*c*^ (μg/mL)
MP196	RWRWRW-NH_2_	C_51_H_69_N_19_O_6_	1044.24	+3	12.30	30.275	99.15	>64
WR-6	WRWRWR-NH_2_	C_51_H_69_N_19_O_6_	1044.24	+3	12.30	34.157	95.31	>64
KW-6	KWKWKW-NH_2_	C_51_H_69_N_13_O_6_	960.20	+3	10.30	31.903	95.98	>64
WK-6	WKWKWK-NH_2_	C_51_H_69_N_13_O_6_	960.20	+3	10.30	25.517	98.83	>64
WL-9	WRLWRLWRL-NH_2_	C_69_H_102_N_22_O_9_	1383.72	+3	12.30	35.818	95.59	8
WV-9	WKVWKVWKV-NH_2_	C_66_H_96_N_16_O_9_	1257.60	+3	10.30	32.456	99.22	>64
WR-12	WRLRWRLRWRLR- NH_2_	C_87_H_138_N_34_O_12_	1852.29	+6	12.70	30.329	99.40	2
WRK-12	Ac-WRLRWKTRWRLK-NH_2_	C_87_H_136_N_30_O_14_	1826.25	+6	12.48	36.513	98.79	2
WKK-12	Ac-WKVKWKVKWKVK-NH_2_	C_86_H_134_N_22_O_13_	1684.17	+6	10.70	31.424	95.27	64

*^a^* The isoelectric point (pI) values of derivatives were determined by ExPASy (http://web.expasy.org/compute_pi/). *^b^* Hydrophobicity was calculated by the percent of acetonitrile in water, 0.1% (*v*/*v*) trifluoroacetic acid (TFA) at HPLC elution. The higher percent acetonitrile, the higher the hydrophobicity. *^c^* Antibacterial activity of peptides against MDR *E. coli* B2 was tested. Four AMPs with antibacterial activity against MDR *E. coli* B2 were highlighted in green.

**Table 2:** Antibacterial spectrum of two amphiphilic peptides against a panel of pathogenic bacteria (MIC or MBC, μg/mL).

Organism and Genotype	WRK-12		WKK-12	AMP	VAN	COL	TIG
	MIC	MBC	MIC				
**Gram-positive bacteria**							
*S. aureus* ATCC 29213	4	4	128	0.25	0.5	16	0.125
MRSA T144	2	2	64	32	1	128	2
*S. aureus* 215 (*cfr* + LZD^R^)	2	2	64	64	1	64	1
*E. faecalis* A4 (VRE)	4	8	64	32	>128	128	0.125
**Gram-negative bacteria**							
*E. coli* ATCC 25922	4	8	128	8	128	0.5	0.125
*E. coli* B2 (*mcr-1* + *bla*_NDM-5_)	4	4	>128	>128	128	8	2
*E. coli* B3-1 (*tet*(X4))	2	2	64	>128	64	0.25	32
*E. coli* 1F28 (*tet*(X4))	2	8	64	>128	128	0.25	32
*S. enteritidis* ATCC 13076	4	4	64	8	128	0.25	0.125

ATCC, American Type Culture Collection; VRE: vancomycin-resistant enterococci, LZD^R^: linezolid-resistant. AMP, ampicillin; VAN, vancomycin; COL, colistin; TIG, tigecycline.

**Table 3:** Antibacterial activity of amphiphilic peptides against tigecycline-resistant clinical isolates (MIC, μg/mL).

Clinical Isolates	Origin	WRK-12	WKK-12	Tigecycline
*E. coli* 1N28	Nasal swab	8	64	32
*E. coli* 1N31		16	64	128
*E. coli* 1C1	Dust	4	64	32
*E. coli* 1F16	Feces	4	64	>64
*E. coli* 1F31		4	64	32
*E. coli* 1A34	Anal swab	2	64	16
*E. coli* 2A19		16	128	8
*E. coli* 2W25	Water	4	64	64
*Shigella* 1F25	Feces	8	64	8

**Table 4:** Salt and serum stability of two amphiphilic peptides (MIC, μg/mL).

Strains	WRK-12	WKK-12
***E. coli* 1F28**	2	64
+Na^+^ (10 mM)	2	>128
+K^+^ (10 mM)	2	64
+Ca^2+^ (10 mM)	8	>128
+10% Serum	4	>128
+10% DMEM	4	>128
*E. coli* 1A34	2	64
+Na^+^ (10 mM)	2	>128
+K^+^ (10 mM)	2	64
+Ca^2+^ (10 mM)	4	>128
+10% Serum	4	>128
+10% DMEM	4	>128

## Supplementary Materials

The following are available online at https://www.mdpi.com/2076-2607/8/9/1398/s1, Table S1: Bacterial strains used in this study; Figure S1: HPLC spectra of engineered antimicrobial peptides on the basis of MP196; Figure S2: MS spectra of engineered antimicrobial peptides on the basis of MP196.
