# Supplementary material for: Potent Broad-Spectrum Antibacterial Activity of Amphiphilic Peptides against Multidrug-Resistant Bacteria
Source: Microorganisms. 2020 Sep 11;8(9):1398. doi: 10.3390/microorganisms8091398 (PMC7564829; doi:10.3390/microorganisms8091398)
Supplement: Supplementary file 1 [file microorganisms-08-01398-s001.pdf]

Supplementary material

Table S1 Bacterial strains used in this study.

Organisms and phenotypes	Sources
**Gram-positive bacteria**	
S. aureus 29213	ATCC
MRSA T144	In this study
S. aureus 215 (LZD^R + *cfr*)	In this study
E. faecalis A4 (VRE, VanA)	In this study
**Gram-negative bacteria**	
E. coli 25922	ATCC
S. enteritidis 13076	ATCC
E. coli B2 (*mcr-1* + bla_{NDM-5})	In this study
E. coli B3-1 (*tet*(X4))	In this study
E. coli 1F28 (*tet*(X4))	In this study
E. coli 1N28 (*tet*(X4))	In this study
E. coli 1N31 (*tet*(X4))	In this study
E. coli 1C1 (*tet*(X4))	In this study
E. coli 1F16 (*tet*(X4))	In this study
E. coli 1F31 (*tet*(X4))	In this study
E. coli 1A34 (*tet*(X4))	In this study
E. coli 2A19 (*tet*(X4))	In this study
E. coli 2W25 (*tet*(X4))	In this study
Shigella 1F25 (*tet*(X4))	In this study

ATCC, American Type Culture Collection; LZD, linezolid; RIF, rifampicin.

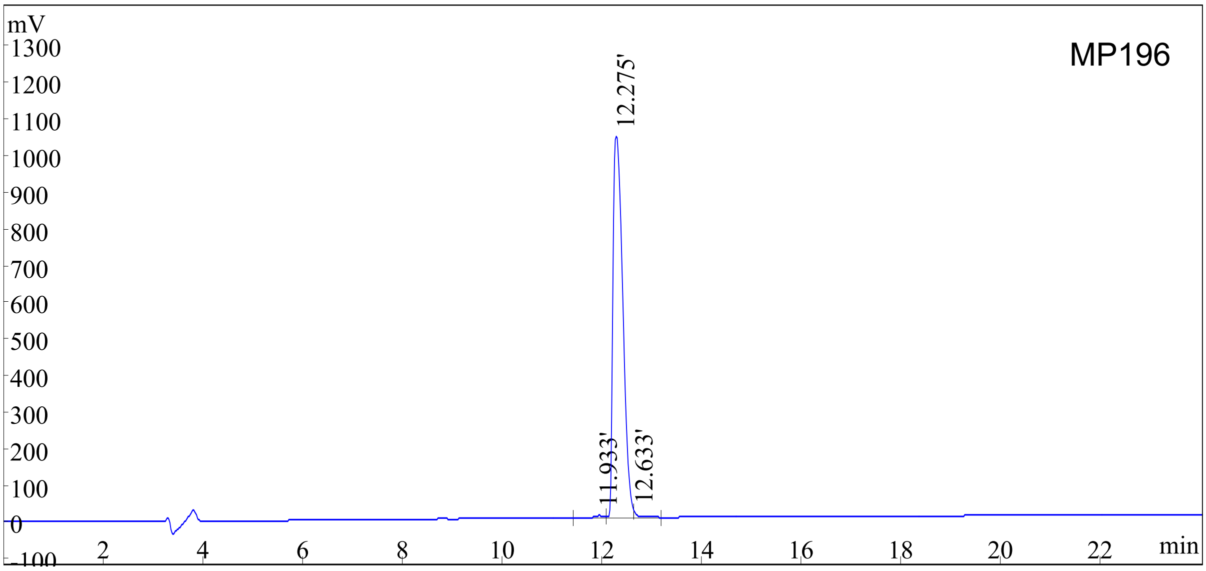
MP196
mV
min
12.275'
11.933'
12.633'

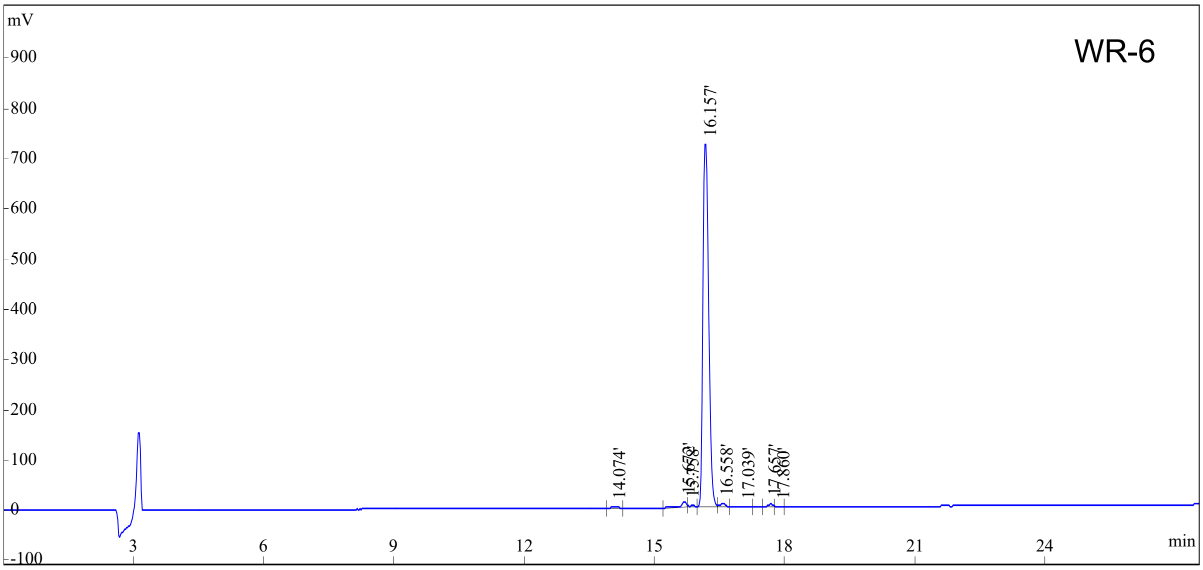
WR-6
mV
min
16.157'
14.074'
16.558'
17.039'
17.860'

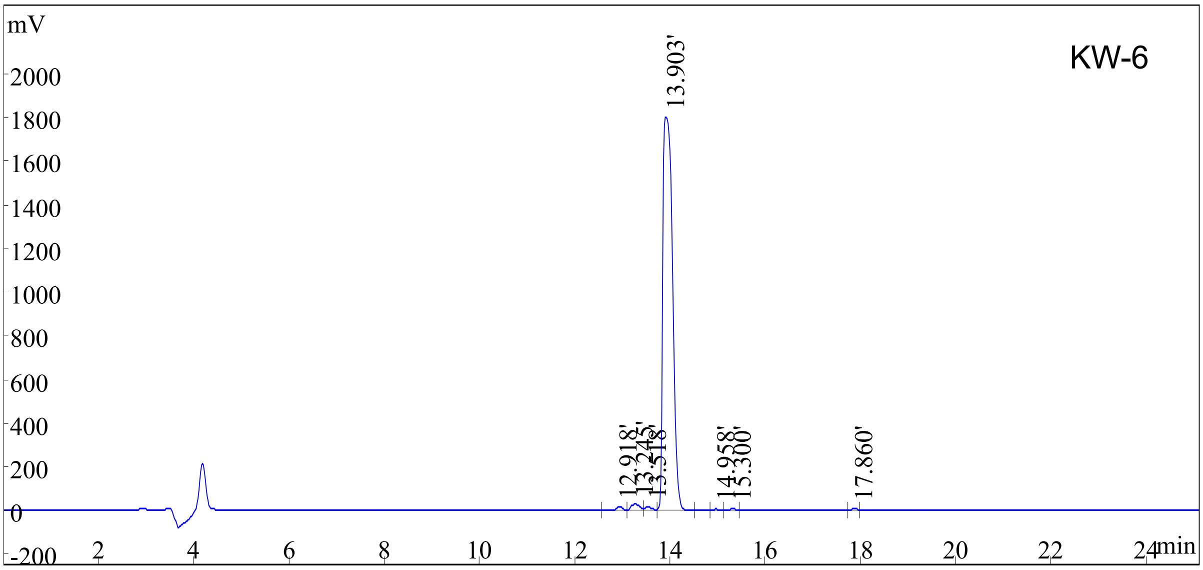
KW-6
mV
min
13.903'
12.918'
17.860'

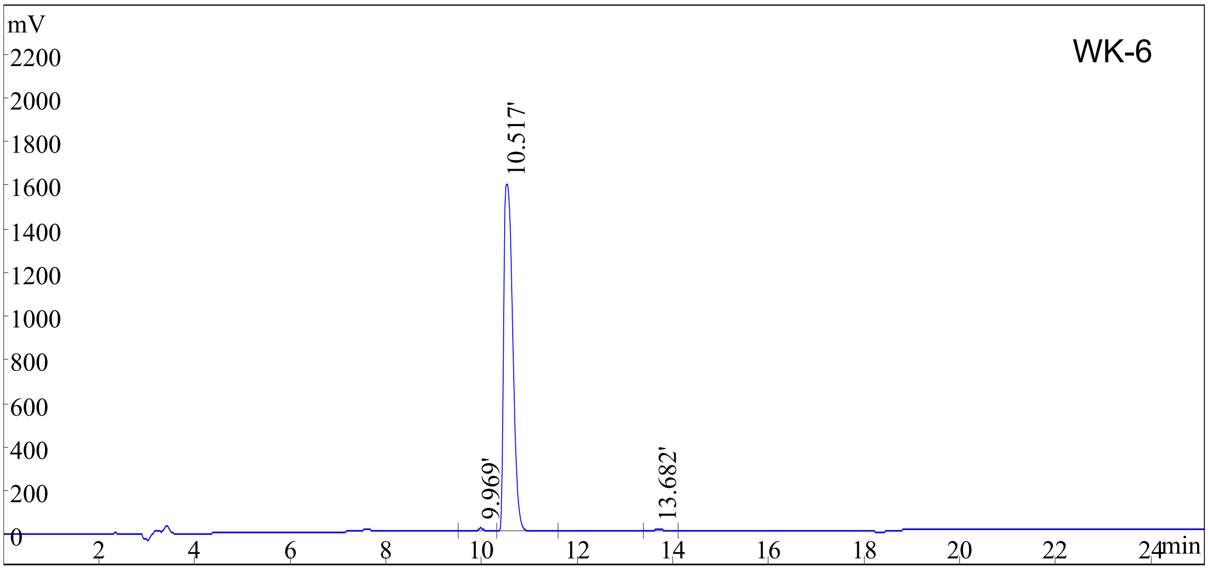
WK-6
mV
2200
2000
1800
1600
1400
1200
1000
800
600
400
200
0
10.517'
9.969'
13.682'
2
4
6
8
10
12
14
16
18
20
22
24
min

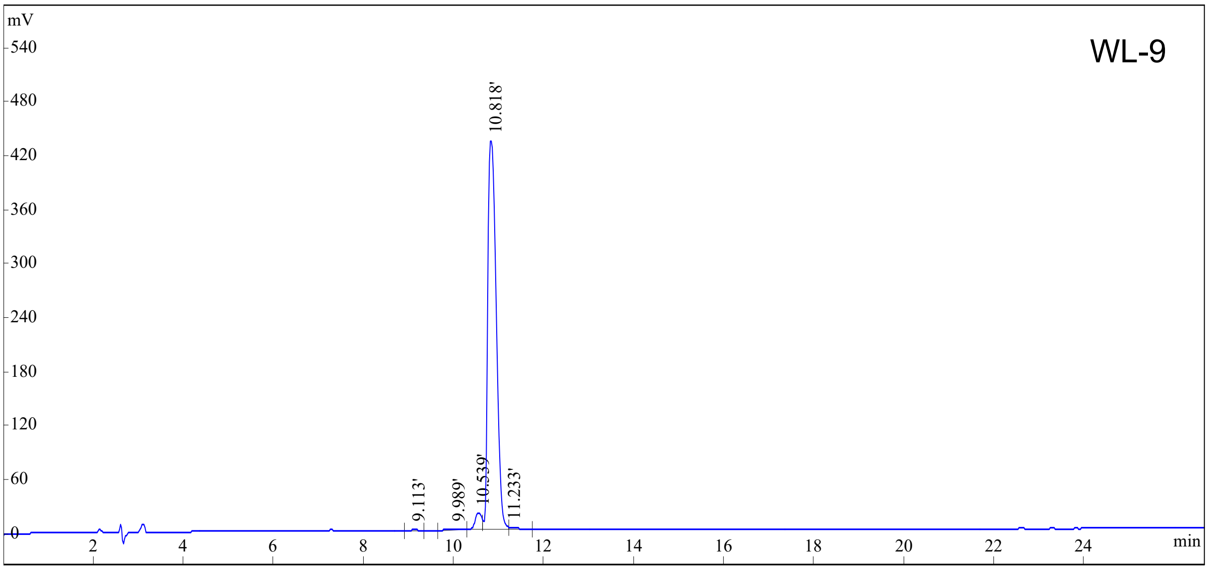
WL-9
mV
540
480
420
360
300
240
180
120
60
0
10.818'
9.113'
9.989'
10.539'
11.233'
2
4
6
8
10
12
14
16
18
20
22
24
min

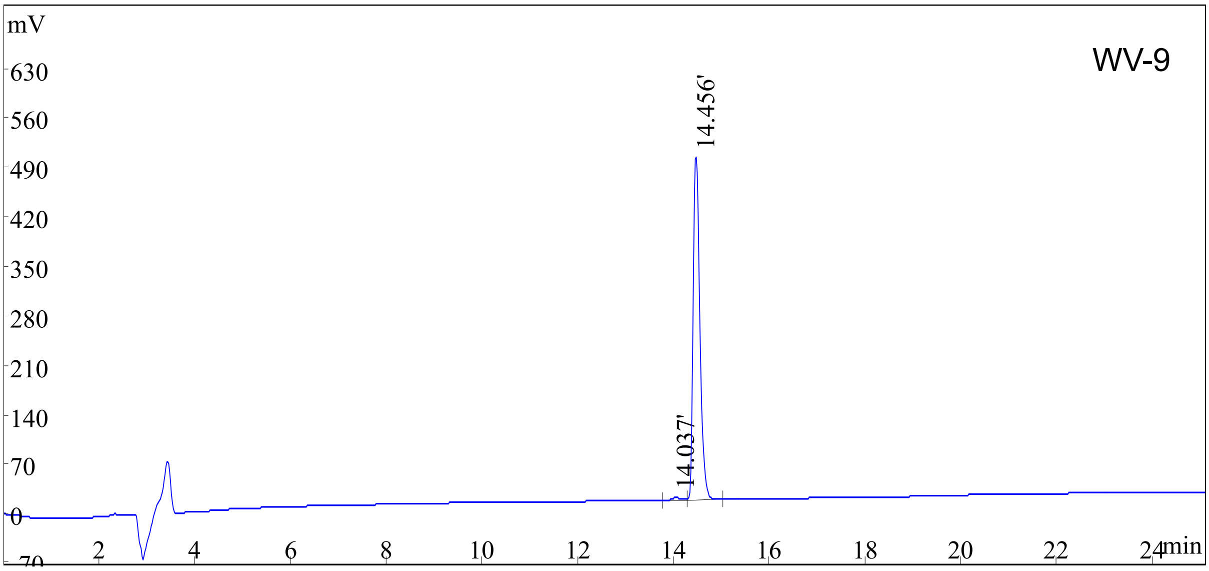
WV-9
mV
630
560
490
420
350
280
210
140
70
0
-70
14.456'
14.037'
2
4
6
8
10
12
14
16
18
20
22
24
min

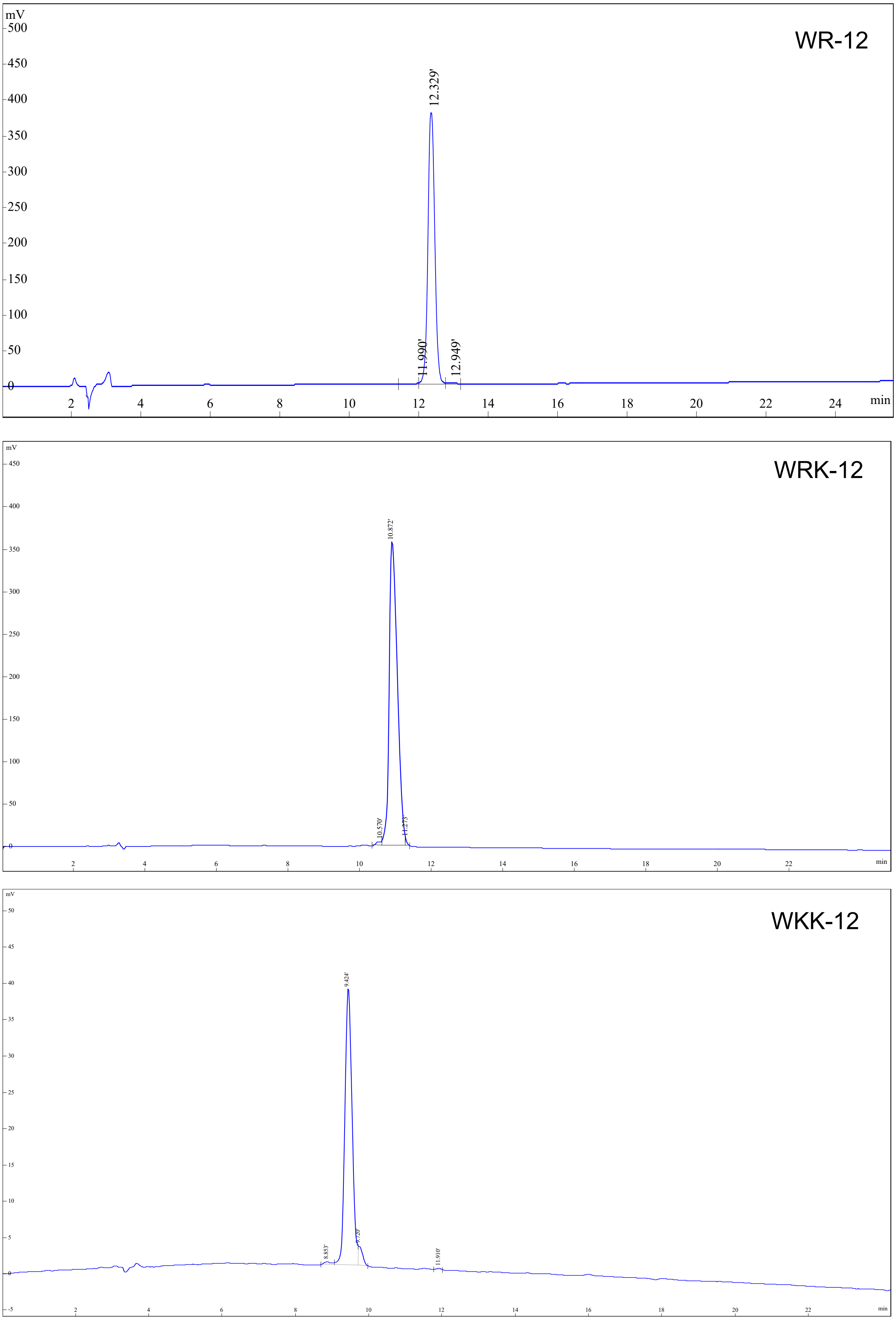

Figure S1 HPLC spectra of engineered antimicrobial peptides on the basis of MP196.

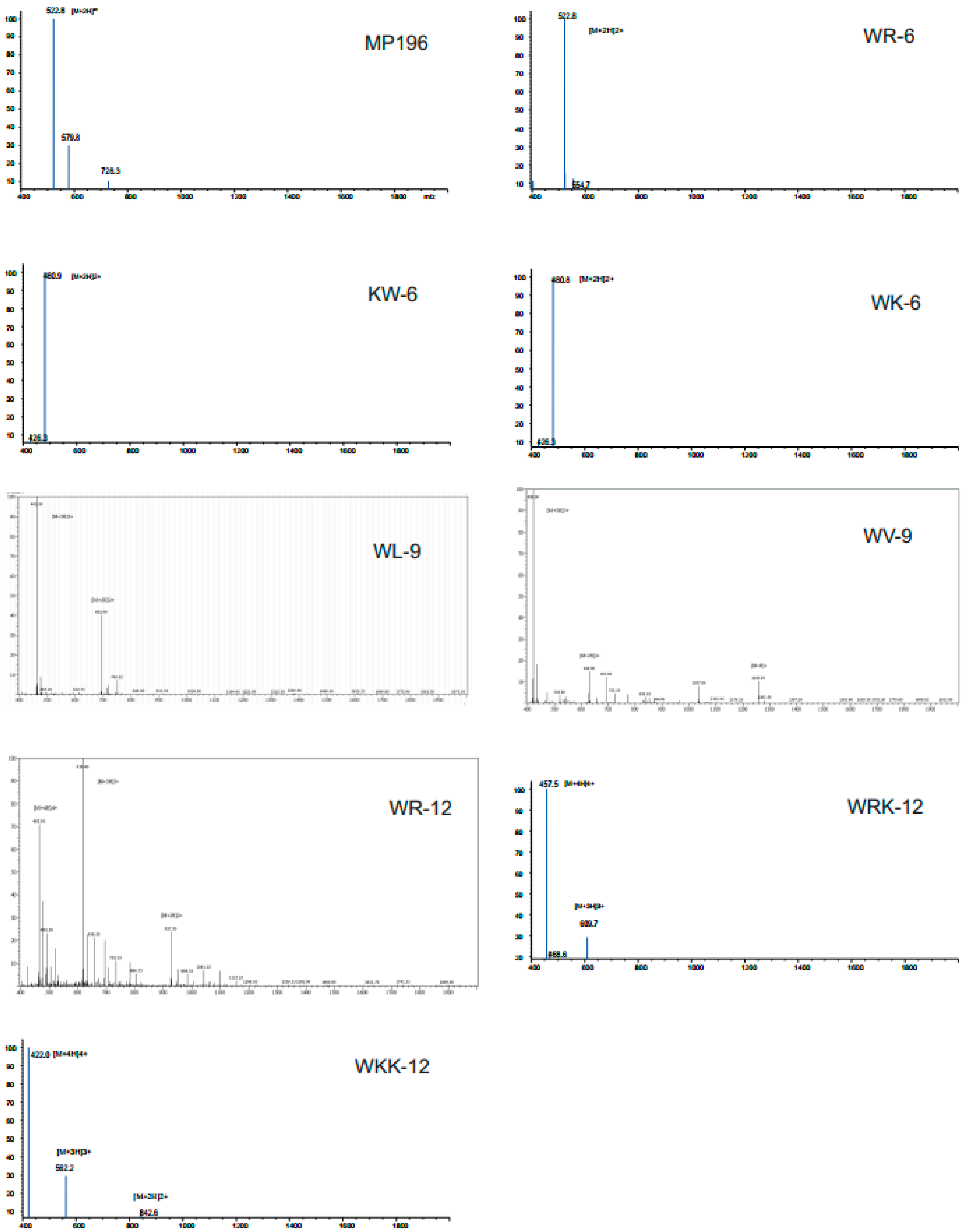

Figure S2 MS spectra of engineered antimicrobial peptides on the basis of MP196.
